# Respiratory Symptoms and Changes of Oxidative Stress Markers among Motorbike Drivers Chronically Exposed to Fine and Ultrafine Air Particles: A Case Study of Douala and Dschang, Cameroon

**DOI:** 10.3390/jcm13133816

**Published:** 2024-06-28

**Authors:** Joseph Eloge Tiekwe, Nadine Ongbayokolak, Solange Dabou, Cerge Kamhoua Natheu, Marie Stéphanie Goka, Prosper Cabral Nya Biapa, Isabella Annesi-Maesano, Phélix Bruno Telefo

**Affiliations:** 1 Department of Biochemistry, University of Dschang, Dschang P.O. Box 67, Cameroon; nongbayokolak@yahoo.fr (N.O.); solangedabs@gmail.com (S.D.); cergenk@gmail.com (C.K.N.); stephy_vidoq@yahoo.fr (M.S.G.); prbiapa@yahoo.fr (P.C.N.B.); bphelix@yahoo.co.uk (P.B.T.); 2 Division of Pharmacology and Toxicology, Faculty of Medicine, University of Leipzig, 04109 Leipzig, Germany; 3 Desbrest Institute of Epidemiology and Public Health, University Montpellier, INSERM, 34090 Montpellier, France; isabella.annesi-maesano@inserm.fr; 4 Division of Respiratory Medicine, Allergology, and Thoracic Oncology, University Hospital of Montpellier, 34295 Montpellier, France

**Keywords:** urban air pollution, respiratory symptoms, oxidative stress, motorbike drivers, sub-Saharan Africa

## Abstract

Recent studies revealed that the high production of reactive oxidative species due to exposure to fine or ultrafine particles are involved in many chronic respiratory disorders. However, the poor standard of clinical data in sub-Saharan countries makes the assessment of our knowledge on the health impacts of air pollution in urban cities very difficult. **Objective:** The aim of this study was to evaluate the distribution of respiratory disorders associated with exposure to fine and ultrafine air particles through the changes of some oxidative stress biomarkers among motorbike drivers from two cities of Cameroon. **Methods:** A cross-sectional survey using a standardized questionnaire was conducted in 2019 on 191 motorcycle drivers (MDs) working in Douala and Dschang. Then, the activities of superoxide dismutase (SOD) and the level of malondialdehyde (MDA) were measured using colorimetric methods. The data of participants, after being clustered in Microsoft Excel, were analyzed and statistically compared using SPSS 20 software. **Results:** The motorbike drivers recruited from both cities were from 21 to 40 years old, with a mean age of 29.93 (±0.82). The distribution of respiratory disorders, such as a runny nose, cold, dry cough, chest discomfort, and breathlessness, was significantly increased among MDs in Douala. According to the results of biological assays, SOD and MDA were significantly greater among the MDs recruited in Douala compared to those of Dschang. The change in these oxidative stress markers was significantly positively correlated with the mobilization of monocytes and negatively correlated with neutrophils, showing the onset and progression of subjacent inflammatory reactions, and it seemed to be significantly influenced by the location MDs lived in. **Conclusions**: Through this study, we have confirmed the evidence supporting that the onset and progression of oxidative stress is caused by the long-term exposure to fine or ultrafine air particles among working people living in urban cities. Further studies should be conducted to provide evidence for the cellular damage and dysfunction related to the chronic exposure to fine particulate matter (PM) in the air among working people in the metropolitan sub-Saharan Africa context.

## 1. Introduction

**Figure 1:**
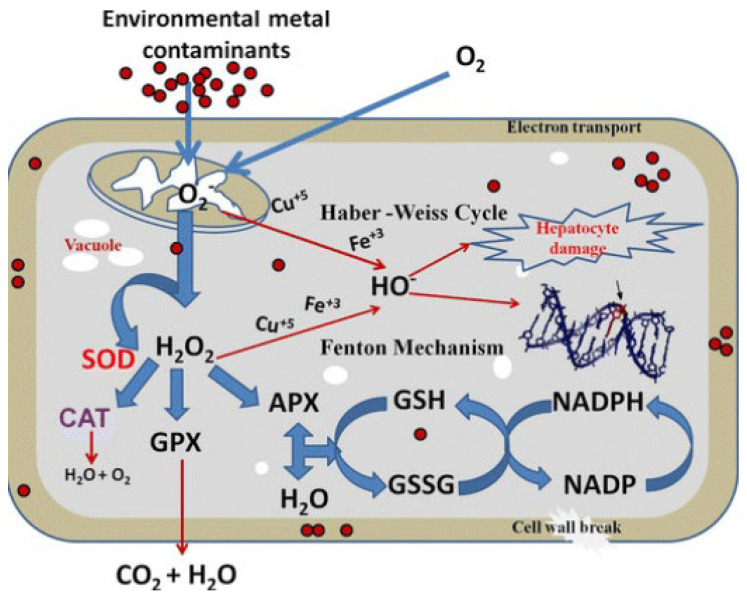
Cellular oxidative stress mechanism and antioxidant defense system. Superoxide dismutase (SOD), catalase (CAT), glutathione (GSH), oxidized glutathione (GSSG), glutathione peroxidase (GPX), ascorbate peroxidase (APX), nicotinamide adenine dinucleotide phosphate (NADPH), and hydrogen peroxide (H_2_O_2_) [13].

Fine and ultrafine air particles are investigated according to the recent studies on acute and chronic respiratory diseases. Among the physiological mechanisms triggering these pulmonary dysfunctions, oxidative stress with inflammation has been revealed as one of main mechanisms, which can be a risk factor of cardiovascular and neurological diseases. Still, the evidence is unclear. However, further studies are being carried out, aiming to confirm it. The chronic exposure to fine and ultrafine urban PM in the air leads to an increase in reactive oxidative species in our cells and tissues, triggering many oxidative and inflammatory biomarkers [1]. Epidemiological studies generally show that fine and ultrafine particles (PM_2.5_, PM_0.1_) have a greater impact on our health than coarse PM_10_ fractions [2,3]. Fine and ultrafine ambient urban particles are mostly derived from the combustion processes of urban traffic, some industrial activities, and consist of carbonaceous particles with associated absorbed organic compounds and polycyclic aromatic hydrocarbons, as well as reactive metals, such as iron, copper, nickel, zinc, and vanadium. The chemical compositions and nanostructures of air pollutants have been recently added to enhance the traditional classification of air pollution, which is based on particle size and mass concentration [4,5]. These air particles, once in contact with the lung’s epithelium, initiate inflammatory mechanisms, activate macrophages, modulate gene expression, and activate transcriptions factors [6], which can lead to the development of chronic respiratory diseases. The understanding of oxidative molecules based on fine or ultrafine air particles, their transmission to the target organ, and the molecular pathways generating “reactive oxygen species” (ROS) in physiological and pathological processes (Figure 1) are the areas focused on in experimental studies [7]. 

Many inhalation studies on experimental animals have revealed adverse effects associated with the triggering of ROSs, which depend on the emission source and composition of particles [5]. Recent studies showed that ROSs are the most important mediators of particle toxicity, with a notable association with respiratory diseases. Ultrafine and fine particles contain some ROSs, as well as redox-active components, which can lead to ROS generation upon the interaction with biological and cellular components [2,5]. Therefore, ROSs are produced during a cell’s metabolic process and they are usually generated from photolysis, radiolysis, and hemolytic fission, where chemical bonds break. Moreover, each newly created fragment preserves one of the bounded initial electrons [5]. Overall, ROSs contribute to many different cellular processes in organisms, such as protein phosphorylation, secondary messengers, activation of transcriptions factors, immune responses, and apoptosis [5,6]. Moreover, mitochondria are involved in the endogen generation of ROSs through the electron transport chain, which produce the primary ROS, the superoxide anion radical (O_2_^−^). This radical can interact with other molecules to generate secondary ROSs, such as hydrogen peroxide (H_2_O_2_) and the hydroxyl radical (HO*), which leads to cellular and molecular damage through lipid peroxidation, notably when the formation of ROSs is used for antioxidant defense [5].

Indeed, cells from organisms possess an antioxidant defense system that consists of the following enzymes—superoxide dismutase (SOD), catalase (CAT), and glutathione peroxidase (GPx)—and some non-enzymatic antioxidants—such as reduced gluthatione (GSH) and vitamins A, C, and E [8,9]. These antioxidants help the organism to tackle the deleterious effects of ROSs and decrease cellular damage (Figure 1). SOD is one the first lines of protection against ROSs [9]. It catalyzes the breakdown of the superoxide anion (O_2-_) into the less potent oxidant hydrogen peroxide (H_2_O_2_) and allows, in combination with the action of CAT, to abate the production of superoxide radicals and hydrogen peroxide, consequently protecting cellular components against ROSs [10]. Furthermore, lipid peroxidation has been commonly used as an indicator of ROS-mediated damage to cell membranes. Malondialdehyde (MDA) is one of the best-studied end products of the peroxidation of polyunsaturated fatty acids in clinical samples, and it is frequently used to estimate oxidative stress conditions [11]. It is a lipid peroxidation marker widely utilized to estimate the cell damage induced by ROSs [12].

Oxidative toxic stress in the cells of an organism is also evaluated by measuring the total antioxidant capacity (TAC) and total oxidant status (TOS), which both reflect the oxidative and antioxidative status of the body [14]. When recent studies investigated the association between human exposure to air pollution and oxidative stress, they considered the antioxidative defense status or ROS levels among people in polluted areas by using oxidative stress biomarkers (such as MDA, SOD, CAT, and GSH) [15,16]. Although the cause of significantly poor air quality is accelerated in urban development and rapid population growth, there are still very few studies today that have revealed the adverse effects of how urban air pollution causes changes in oxidative stress parameters among people who are exposed to it [5]. In sub-Saharan Africa, the data that explore or indicate the triggering of oxidative stress biomarkers from the chronic exposure to ambient, fine, or ultrafine air particles in the urban parts of the world are almost nonexistent [17]. 

In this view, we carried out a survey in two towns of Cameroon, Douala and Dschang, between February and July 2019 to investigate the possible oxidative and pro-inflammatory responses of pulmonary human cells chronically exposed to fine or ultrafine urban air particles through an examination of the activity of superoxide dismutase (SOD) and levels of malondialdehyde (MDA) among motorbike drivers chronically exposed to different levels of urban pollution.

## 2. Materials and Methods

### 2.1. Study Area

**Figure 2:**
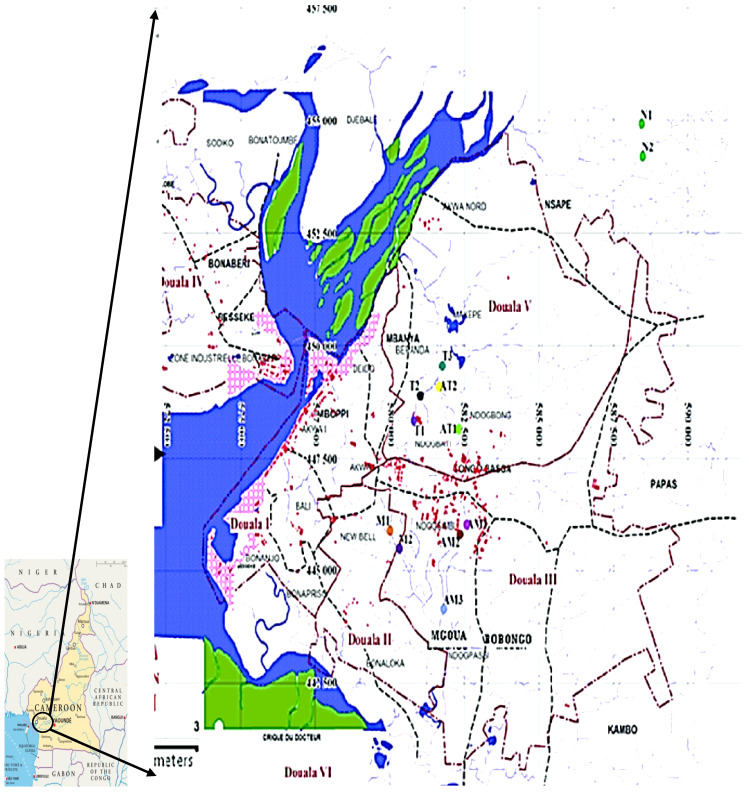
Localization of Douala and its subdivisions [19].

This cross-sectional study was conducted in the urban areas of Douala and Dschang. Douala, the economic capital of Cameroon which has more than 3.5 million inhabitants, is the highest dynamically dense city and one of the largest business and economic hubs of Central Africa [18]. It has been separated into five subdivisions: from Douala I to Douala V (Figure 2). Douala I and II are near the ports and administrative centers. Commercial and trade zones were also created. Douala III, IV, and V are popular sites that are outside the city’s center. Furthermore, zones IV and V contain the most industrial plants of Douala, some of which are often not far from inhabited areas. Moreover, several intersections in Douala are subjected to huge daily urban traffic, with thousands of vehicles and motorcycles, which are sometimes second-hand and dilapidated. 

On the other hand, Dschang, which is within the Menoua Division in the West Region of Cameroon, is situated about 216.3 km from Douala at 1500 m above sea level. It is an agricultural and rural town in which industrial activity is almost nonexistent [20]. 

### 2.2. Target Population and Sampling

Subjects recruited from both cities were MDs with an average age of thirty years. Dschang City was considered the control zone because urban traffic and industrial activity within it were lower than in Douala; thus, this city could was considered to be less polluted than Douala. MDs were mostly recruited from the points at which they were stationed (near large crossroads) and were called to respond to an interviewer-administered questionnaire, which contained questions on their daily level of general sensitivity regarding discomfort related to air pollution, and they were invited to standard hospitals for testing. In this view, most of our attention was paid to how long they work during the day, and their places of residence were also considered. Their blood samples were taken and analyzed according to Good Laboratory Practice (GLP) guidelines. In addition to the analysis of hematological data, (white blood cells and platelets), activity of superoxide dismutase (SOD) and level of malondialdehyde (MDA) were tested. Some confounding factors, such as tobacco usage and frequent alcohol consumption, were considered.

#### 2.2.1. Eligibility Criteria 

Healthy MDs permanently living or working for at least five years near the studied regions with bad air quality were included in this study. Signed informed consent was obtained from the MDs prior to inclusion.

MDs under treatment against chronic respiratory and cardiovascular diseases, hepatitis, and kidney ailments, as well as MDs who currently have or with a history of diabetes and others chronic metabolic disorders were excluded from the sample because of possible modifications of the targeted inflammatory and oxidative stress markers induced by these medical conditions. 

#### 2.2.2. Blood Collection and Measurement

The blood of the motorbike drivers from both cities were taken from a five-milliliter sample of coagulated blood and EDTA tubes at the health examination centers in Douala and Dschang. These blood specimens were centrifuged at 3000 rpm for 10 min within 45 min of collection. Then, serums were aliquoted, coded, and stored at −80 °C for an assessment of studied biological parameters.

#### 2.2.3. Assessment of Hematological and Oxidative Stress Biomarkers 

Oxidative stress effects of ambient air pollution in this study were evaluated through an assessment of superoxide dismutase (SOD) and malondialdehyde (MDA). Then, the mobilization of blood cells through a short- or long-term inhalation of fine and ultrafine urban air particles was evaluated by measuring the hematological parameters.

- **Measurement of hematological markers**

Total red blood cell (RBC) count (×10^6^/μL), hemoglobin (Hb) content (g/dL), total white blood cell (WBC) count (×10^3^/μL), and platelet count (×10^3^/μL) were assessed using a Urit 2900 plus hematology analyzer (Urit Medical Electronics Co., Ltd, Shenzhen, China).

- ***Measurement of MDA***

MDA is a biproduct of lipid peroxidation. It was measured using a colorimetric method using thiobarbituric acid reactive substances (TBARSs). Indeed, serum specimens were mixed with a solution containing 1% thiobarituric acid, 1% acetic acid solution, and distilled water, and they were heated for 60 min in a boiling water bath. The mixture was then placed in cold water to reach room temperature. Subsequently, TBARS adducts were extracted by n-butanol, followed by centrifugation at 3500 g for 10 min. The layer, including n-butanol, was removed, and the absorbance of the solution was measured at 532 nm [21].

### 2.3. Measurement of SOD Activity

SOD is one of the most important antioxidant enzymes. It catalyzes the dismutation of superoxide anions (O^2−^) to generate oxygenated water (H_2_O_2_) and molecular oxygen (O_2_). It was determined by the Misra and Fridovich method (1972) and based on SOD’s ability to inhibit or delay the self-oxidation of adrenaline in adrenochrome in a basic environment (pH 10.2). The increase in the absorption reading to 480 nm was proportional to the activity of superoxide dismutase. In total, 150 µL of each test solution (serum) and prepared controls was introduced into the test tubes with 500 μL of carbonate-bicarbonate buffer (pH 10.2; 0.3 M; pKa 10.3), 250 μL of an EDTA solution (0.6 mM), and 350 μL of distilled water. The resulting mixture was homogenized, and 250 μL of an epinephrine solution (4.5 mM) was added to initiate the reaction. Optical density was read after 30 s and 180 s at 480 nm [22].

### 2.4. Statistical Analyses

After being evaluated, data were classified, checked, and analyzed through Statistical Package for Social Sciences (SPSS) version 20.0. Frequencies and mean ± standard deviation (sd) were considered as most important parameters to assess the comparison of health effects of fine and ultrafine urban particles between subjects from Douala and Dschang. Both of these parameters were computed where appropriate. Statistical tests, such as Pearson’s independent chi-squared test and Fisher’s exact test, were used. The means of the obtained parameters from the motorbike drivers of both cities were statistically differentiated through Student’s *t*-test, and the correlation between hematological parameters and oxidative stress markers was assessed using Pearson’s test. The effects of place of residence, time of exposure, age, level of education, smoking status, alcohol consumption, fuel used for cooking meals, BMI, and the distribution of observed symptoms on changes in oxidative stress markers were assessed. The models were adjusted for these different factors. The oxidative stress biomarker levels were normally distributed. The significance was set at *p*-values of <0.05 and <0.001.

## 3. Results

### 3.1. Demographic Characteristics of Subjects

Table 1 presents the basis socio-demographic characteristics of the MDs recruited in both of the studied cities. The majority of MDs were young, with age varying between 21 and 39 years. The mean age of the participants of this study was 29.93 (±0.82) and was approximately the same in both cities. The MDs in Douala were significantly more overweight than those recruited in Dschang (3.612 (*p* < 0.001)), according to the body mass index (BMI). 

The majority of MDs recruited in both cities reached secondary school education (65.96%), with a higher percentage in Douala. Most of them lived in Douala III, one of Douala’s most populous neighborhoods. About 97% recruited in this town work in the entire city. 

As presented in Table 2, the majority of MDs recruited in both cities stated that they never smoked (97.90%) but drink alcohol regularly (91.09%), with a higher percentage among those from Douala. The majority of these have work experience ranging from 7 to 14 years (73.82%) and an average working time of 8 to 20 h per day (85.86%).

### 3.2. Distribution of Symptoms Related to Air Pollution among Participants

According to the data in Table 3, the symptoms associated with upper airway disorders were more distributed among the MDs in Douala than those in Dschang. A runny nose, cold, and sore throat were significantly common among the participants. Moreover, a dry cough, chest discomfort, and breathlessness represented the most common symptoms associated with lower airway disorders, and they were significantly more common among the MDs recruited in Douala than those in Dschang.

As shown in Table 4, the symptoms associated with general discomfort, such as headache, eye irritation, conjunctivitis, general tiredness, and sweating, were significantly more prevalent among the MDs in Douala. Nausea appeared to be significantly more prevalent among the MDs in Dschang.

### 3.3. Biological Assays

Biological data obtained from the MDs, including hematological parameters and oxidative stress markers, such as malondialdehyde and SOD, are presented in Table 5. As shown, the neutrophils in the MDs in Douala appear to be significantly lower than those of the MDs from Dschang. The monocyte count was significantly higher among the MDs in Douala. Differences between both categories of participants for changes in lymphocytes were not significant. On the other hand, the hematocrit was significantly higher among the MDs in Douala than those of Dschang. 

**Figure 3:**
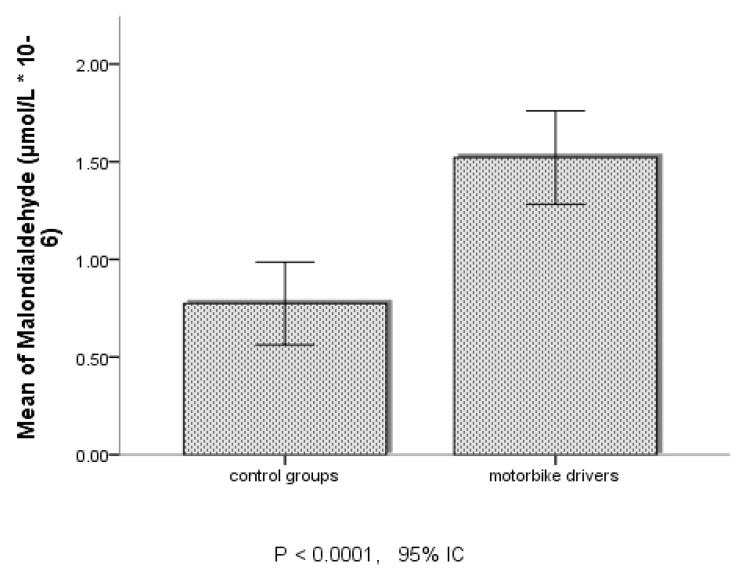
Serum levels of MDA between control group and MDs in both cities. IC: confidence interval.

**Figure 4:**
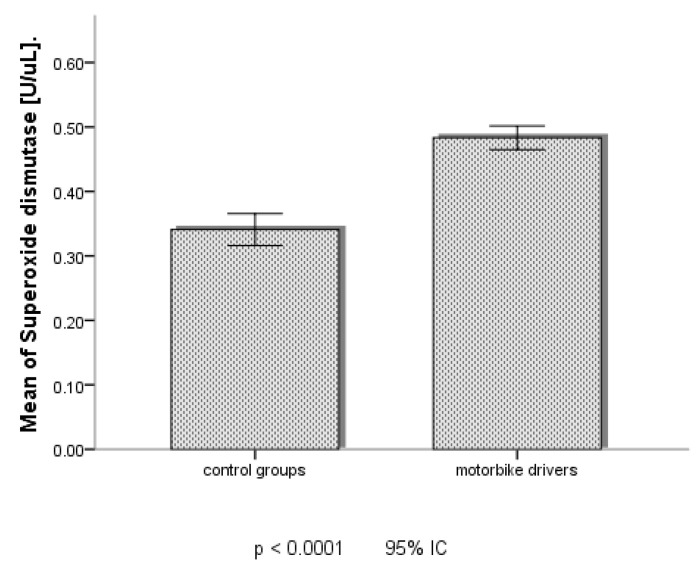
Serum level of SOD between control group and MDs in Douala. IC: confidence interval.

According to the data of oxidative stress markers in Table 5 and Figure 3 and Figure 4, MDA and SOD seemed to be significantly higher among the MDs in Douala than those of Dschang.

### 3.4. Changes in Hematological Parameters and Oxidative Stress Markers in Motorbike Drivers in Douala

As shown in Table 6, a significant decrease in neutrophils with the changes in SOD and MDA was observed. Monocytes increased significantly with the variations in both oxidative stress makers. Correlations between changes in oxidative stress and others hematological parameters, such as red blood cells, hemoglobin, hematocrit, lymphocytes, and platelets, were not observed.

### 3.5. Exposure Factors and Changes in Oxidative Stress Markers in Motorbike Drivers in Douala

The data from Table 7 revealed a significant correlation between the place of residence and changes in SOD and MDA. Both of these oxidative stress markers seemed to not be influenced by MDs’ work experience (in years) and their average working time (in hour per day). 

As shown in Table 8, significant changes in MDA and SOD were observed regarding MDs’ place of residence after an adjustment for age, level of education, body mass index (BMI), alcohol, and the use of domestic gas, firewood, and coal. Other exposure factors were not observed to have an influence, even after an adjustment.

### 3.6. Respiratory Disorders and Changes in Oxidative Stress Markers among Motorbike Drivers in Douala

As shown by the results in Table 9, a significant correlation between the prevalence of a cold and changes in oxidative stress markers, SOD, and MDA was observed among the MDs. The prevalence of other symptoms, such as sinusitis, runny nose, current fever, and sore throat, was not significantly correlated with the changes in both oxidative stress markers.

However, the prevalence of a dry cough seemed significantly correlated with the changes in MDA. No significant correlation was found between the prevalence of wheezing, chest discomfort, and variations in the oxidative stress markers among the MDs. The prevalence of breathlessness was almost significantly correlated with the changes in SOD.

### 3.7. General Discomfort and Changes in Oxidative Stress Markers among Motorbike Drivers in Douala

It can be observed from Table 10 that there was an absence of a significant correlation between the prevalence of a headache, eye irritation, sweating, dizziness, conjunctivitis, general tiredness, nausea, and changes in both oxidative stress markers among the MDs.

## 4. Discussion

The results of this study show how short- or long-term exposure in polluted sites in urban areas could lead to the development of acute or chronical respiratory disorders and the mobilization and accumulation of ROSs and inflammatory cells, such as white cells. This could lead to the development of cardiovascular diseases among the motorbike divers living and working in these urban areas. The assessment of some biological markers, such as MDA and SOD, revealed the roles of oxidative stress and proinflammatory mechanisms as the main pathways in the onset and progression of theses pathologies. Indeed, the inhalation of fine and ultrafine particulate matter at a cellular level shows a set of mechanisms responsible for the productions of ROSs in the lungs. Some reactions, such as Fenton reactions, try to collapse ROSs, with aim to reduce the increase of ROSs at a cellular level. The Fenton reaction is a complex reaction that causes the decomposition of hydrogen peroxide (H_2_O_2_) to produce hydroxyl radicals (^•^OH, ^•^HO_2_^•^), which are catalyzed by ferrous ions (Fe^2+^/Fe^3+^). These ferrous ions can react and oxidize with various organic compounds, such as carboxylic acids, alcohols, and esters, and transform into inorganic forms (some of which may sometimes come from our diets and our places of residence), resulting in significant oxidation effects [23].

Thus, it was important for us to confirm the evidence that the chronic inhalation of fine and ultrafine pollutants in urban areas can biologically lead to the cellular production of reactive oxygen species, and that the Fenton reaction activates cumulative compounded peroxidation mechanisms with the onset and propagation of oxidated or ionized free radical molecules, which often results in many dangerous processes being initiated with regard to our biological compounds, such as proteins, lipids, DNA, or cells. MDA evaluated in this study is one of the specific and sensitive biomarkers which allowed use to show this molecular aspect among exposed subjects. However, these free radical chain reactions that evolve restrict or control various processes, either partly by compartmentation or by antioxidant defense, and the production of enzyme systems, which can potentially reduce the intracellular concentration of reactive oxygen species. Among the enzyme systems is SOD, which is involved in the reduction of the superoxide anion free radical into molecular oxygen and hydrogen peroxide (H_2_O_2_), which can lead to a decrease in O_2_ -levels, that may cause huge damage to cells. The group of cells called metalloenzymes, as SOD is found in all biological compartments and forms the front lines of defense against reactive oxygen species [24,25]. The biological changes in MDA and SOD evaluated among motorbike drivers confirm the evidence that daily exposure to polluted urban air comprising fine and ultrafine particles leads to advanced free radical cell damage, particularly regarding our pulmonary cells. The facts appear to be more pronounced among the motorbike drivers of Douala than those of Dschang. The results of these clinical observations of oxidative stress with exposure to fine or ultrafine particles corroborate the results of Rezaei et al., 2020, who revealed a greater change in blood MDA among healthy bus drivers, taxi drivers, policeman, and urban service workers exposed to outdoor air pollution in Iran [8]. The same evidence was shown by Sorensen et al., 2003 in female students after environmental exposure to air pollutants [26]. Furthermore, our study showed a significant correlation between a change in MDA and the place of residence as well as the prevalence of symptoms of cold and dry cough among the motorbike drivers. The other exposure factors and symptoms were not significantly correlated. These data revealed that motorbike drivers are not only exposed to pollution at the places where they work but also where they live. In future research, it is important to establish the profile of repartition of fine or ultrafine particles in the urban air of Douala according to different areas. The results on the increase in biological SOD among the motorbike drivers in this study corroborate those of Davel et al., 2012 on rats, which showed that exposure to PM_2,5_ causes an increase in the protein expression of cu/Zn- and Mn-SOD in the pulmonary artery [27]. However the results of Rezaei et al., 2020 showed a decrease in SOD among the people exposed to polluted areas in Iran [8]. Notably, the magnitude of a person’s antioxidative response depends upon several factors, such as the duration of exposure; ambient, fine, or ultrafine particle concentration or toxicity; and the susceptibility of the subjects to polluted air [28]. A low or acute exposure level to air pollutants may increase the activity of SOD system in the early phase to reduce oxidative damage, while chronic exposure, or highly toxic fine or ultrafine air particles, may lead to the halt of endogenous antioxidant responses [24]. For this reason, a progressive decrease in the activity of the enzymatic SOD system could be observed with chronic exposure to pollution, possibly leading to some endogenous antioxidant responses, such as as NF-E2-related factor-2 (Nrf2), Nuclear factor-κB (NF-κB), NAD(P)H quinone oxidoreductase 1 (Nqo1), glutamate-cysteine ligase modifier subunit (Gclm), activation protein-1 (AP-1), and CREB-binding proteins (CBPs), which are regulated and influenced by redox status and involved in the transcriptional regulation of a wide range of genes that are involved in oxidative stress and cellular response mechanisms [28,29,30].

Furthermore, the reduced activity of SOD, as observed in this study, may also result from a decrease in antioxidants’ resistance or an imbalance due to the accumulation of superoxide anions and hydrogen peroxide and their transition into fine or ultrafine air particles and oxidative metabolites [24]. The progressive loss of SOD activity particularly reflects increased oxidative and nitrative stress and could be observed in asthmatic patients. This evidence suggests that SOD serves as a surrogate marker of oxidative stress, notably in the case of asthma severity [31]. However, in this study, none of the motorbike drivers had asthma. 

Nonetheless, regarding the evidence of oxidative stress being related to the exposure to fine or ultrafine particles observed among the motorbike drivers in this study, a change in neutrophils and monocytes was also observed. Clinically, according to previous studies, the inflammatory effects of PM_10_ have been revealed in experimentally studied animals, who underwent a direct insertion of PM_10_ into their lungs to provoke airway inflammation via the release of mediators that exacerbate lung disease in susceptible individuals [31,32]. Moreover, fine and ultrafine particles have been shown to be involved in the direct stimulation of macrophages and epithelial cells to produce various inflammatory cytokines, such as TNF-α, TGF-β1, GM-CSF, PDGF, IL-6, and IL-8. 

Many studies confirm the generation of reactive oxygen and nitrogen species in epithelial cells of the airway, with macrophages that are within the lung being reviewed extensively as well [32,33], particularly in subjects with previous chronic diseases [30]. The pathway that reactive oxygen species are involved in and that causes oxidative stress is responsible for acute and chronic lung inflammation [30,33]. The results of some studies suggest that oxidative stress, inflammation, and tissue damage are directly correlated with exposure to fine or ultrafine air particles [28,33,34]. Moreover, this pathway mobilizes white blood cells and biomarkers involved in the mechanism of phagocytosis. Then, an innate and adaptive immune response is induced by alveolar macrophages [35]. The effects are more pronounced in patients with chronic pulmonary diseases [36]. 

An involvement of PM_2.5_ in the systemic inflammatory response through the release of inflammatory cytokines and chemokines from lung immune cells into circulation was also revealed [32,37]. The underlying mechanisms by which pulmonary oxidative stress leads to systemic inflammation in response to air pollution is still not fully understood and should be studied in more depth. Furthermore, the effects of fine or ultrafine air particles on epithelial barrier function and tight junction expression in nasal mucosa in humans have still not been studied. However, what is known is that we lose barrier function our nasal epithelium through a decreased expression of tight junction proteins and an increased expression of proinflammatory cytokines [31], which mobilize inflammatory cells and mediators. PM_10_ and diesel-exhausted particles, which consist of polyaromatic hydrocarbons (hydrophobic molecules), increase the chance of lung inflammation from inhaling allergens or respiratory viral infections by acting as adjuvants. The activity of several cytokines and chemokines was increased in animal experiments and human studies when lymphocytes and macrophages or monocytes were co-stimulated with particulates in the presence of specific allergens [31]. However, according to cellular and molecular mechanisms, some cytokines, such as vascular endothelial growth factor (VEGF), can be used as potential stimulators of permeability and lung neovascularization, particularly in the case of asthma. VEGF plays an important role in the development of airway remodeling and in the activation of many cells, including basophils [38]. Nevertheless, chronic exposure to ultrafine or fine particulate matter remains associated with reduced lung function and increased COPD prevalence [31].

The results of our study corroborate this evidence, especially in complex urban areas, such as Douala and Dschang, in which air could contain a mixture of pollutants or aerosols. Furthermore, the proximity to different sources of pollution could have a greater influence on the changes in blood oxidative stress or inflammatory markers, as indicated by ones of the studied factor—place of residence. This evidence should be further explored through additional studies on the assessment of air pollution in these areas and the influence of each compound found within aerosols on the progression of pollution-related respiratory or cardiovascular and neurotoxic diseases.

## 5. Conclusions

In conclusion, this study carried out at Douala and Dschang revealed that the oxidative stress pathway is the most important indicator in the development of systemic toxicity related to acute or long-term urban air pollution exposure. More recent studies on either human or animals showed that fine or ultrafine are involved in the progression of airway inflammation and increase the prevalence of respiratory disorders. Oxidative cellular damage and reactive oxygen species responsible for these effects could explain the negative health effects induced by fine or ultrafine particles. The variations in the SOD and MDA markers, and white blood cells (such as as neutrophils and monocytes) prove that motorbike drivers in these chosen urban cities are exposed to the risks of contracting chronic respiratory diseases daily, which could chronically lead to more complicated disorders. For this reason, it is crucial to carry out more sensibilization campaigns or studies among working people in urban cities. Moreover, additional studies are needed to explore and clarify the different mechanisms by which fine or ultrafine particles cause adverse health effects.

## Figures and Tables

**Table 1:** Demographic characteristics of subjects.

	MDs (n = 126)	Control Group (n = 65)	Total	x^2^ (*p*-Value)
**Age groups (years), n (%)**				
[21–27]	51 (16.66)	21 (16.66)	72 (37.69)	3.272 (*p* = 0.352)
[28–33]	45 (35.71)	32 (49.23)	77 (40.31)	
[34–39]	23 (18.25)	9 (13.84)	32 (16.75)	
≥40	7 (5.55)	3 (4.61)	10 (5.23)	
**Mean age (±sd)**	29.87 (±5.47)	30.00 (±5.166)	29.93 (±0.82)	0.165 (*p* = 0.869)
**BMI**	26.50 (±4.50)	24.40 (±1.72)	25.7873 (±3.91)	3.612 (*p* < 0.001 **)
**Level of education, n (%)**				
None	4 (3.17)	2 (3.07)	6 (3.14)	0.950 (*p* = 0.813)
Primary	23 (18.25)	15 (23.07)	38(19.89)	
Secondary	86 (68.25)	40 (61.53)	126 (65.96)	
University	13 (10.31)	8 (12.30)	21 (10.99)	
**Place of residence**				
Douala II	3 (2.38)	0 (0.00)		191 (*p* < 0.001 **)
Douala III	67 (53.17)	0 (0.00)		
Douala IV	34 (26.98)	0 (0.00)		
Douala V	22 (17.46)	0 (0.00)		
Others	0 (0.00)	65 (100)		
**Place of work**				
Douala I	4 (3.17)	0 (0.00)		2.108 (*p* = 0.147)
In all regions	122 (96.82)	65 (100)		

Data are presented as frequency (percentage) and mean (±standard deviation). MDs = motorbike drivers. Pearson’s chi-squared test was used to compare proportions. ** Significant difference at a *p*-value less than 0.001.

**Table 2:** Confounding and exposure factors.

	MDs (n = 126)	Control Group (n = 65)	Total	x^2^ (*p*-Value)
**Tobacco smoking, n (%)**				
Ex-smoker	1 (0.79	3 (4.61)	4 (2.09)	0.464 (*p* = 0.793)
Never smoked	125 (99.20)	62 (95.38)	187 (97.90)	
**Alcohol consumption, n (%)**				36.173
Regular use	126 (100)	48 (73.86)	174 (91.09)	(*p* < 0.001 **)
Temporary	0 (0.0)	4 (6.15)	4 (2.09)	
Never	0 (0.0)	13 (20.00)	13 (6.80)	
**Work experience (years), n (%)**				
≤6	7 (5.55)	30 (46.15)	37 (19.37)	45.658
[7–14]	110 (87.30)	31 (47.69)	141 (73.82)	(*p* < 0.001 **)
≥14	9 (7.14)	4 (6.15)	13 (6.80)	
**Average working time (h/day), n (%)**				
≤7	9 (7.14)	18 (27.69)	27 (14.13)	14.918
[8–20]	117 (92.85)	47 (72.30)	164 (85.86)	(*p* < 0.001 **)
≥21	0 (0.0)	0 (0.0)	0 (0.0)	

Data are presented as frequency (percentage) and mean (±standard deviation). MDs = motorbike drivers. Pearson’s chi-squared test was used to compare proportions. ** Significant difference at a *p*-value less than 0.001.

**Table 3:** Distribution of symptoms related to respiratory disorders among subjects.

Different Symptoms Related to Respiratory Disorders	MDs (n = 126)	Control Group (n = 65)	x^2^ (*p*-Value)
**Upper airway symptoms, n (%)**			
Sinusitis	11 (4.54)	6 (9.23)	0.013 (*p* = 0.908)
Runny nose	62 (25.61)	20 (25.00)	5.949 (*p* = 0.015 *)
Cold	108 (44.62)	34 (42.50)	25.090 (*p* < 0.001 **)
Current fever	30 (12.39)	16 (20.00)	0.015 (*p* = 0.902)
Sore throat	31 (12.80)	4 (5.00)	9.752 (*p* = 0.002 *)
**Total**	**242**	**80**	
**Lower airway symptoms, n (%)**			
Dry cough	101 (39.45)	30 (36.14)	23.013 (*p* < 0.001 **)
Wheezing	28 (10.93)	17 (20.48)	0.368 (*p* = 0.544)
Chest discomfort	63 (24.60)	21 (25.30)	5.448 (*p* = 0.020 *)
Breathlessness	64 (25)	15 (18.07)	13.582 (*p* < 0.001 **)
**Total**	**256**	**83**	

Data are presented as frequency (percentage) and mean (±standard deviation). MDs = motorbike drivers. Pearson’s chi-squared test was used to compare proportions. * Significant difference at a *p*-value less than 0.05. ** Significant difference at a *p*-value less than 0.001.

**Table 4:** Distribution of systematic symptoms among subjects.

Systemic Symptoms	MDs (n = 126)	Control Group (n = 65)	x^2^ (*p*-Value)
Headache	103(26.68)	36 (29.03)	15.040 (*p* < 0.001 **)
Dizziness	6 (1.55)	7 (5.64)	2.440 (*p* = 0.118)
Eye irritation	88 (22.79)	26 (20.96)	15.869 (*p* < 0.001 **)
Conjunctivitis	48 (12.43)	14 (11.29)	5.362 (*p* = 0.021 *)
Sweating	60 (15.54)	10 (8.06)	19.190 (*p* < 0.001 **)
General tiredness	74 (19.17)	22 (17.74)	10.621 (*p*< 0.001 **)
Nausea	7 (1.81)	9 (7.25)	3.840 (*p* = 0.051)
**Total**	**386**	**124**	

Data are presented as frequency (percentage). MDs = motorbike drivers. Pearson’s chi-squared test was used to compare proportions for each symptom. * Significant difference at a *p*-value less than 0.05. ** Significant difference at a *p*-value less than 0.001.

**Table 5:** Biological parameters of participants.

Biological Markers	MDs (Mean ± SD)	Group Control (Mean ± SD)	Student’s *t*-Test *p*-Value
**Hematological parameters**		**Reference**	
Red blood cell count (×10^12^/L)	4.9794 ± 0.53334	5.0932 ± 0.53334 (4.5–5.9)	0.330
Hemoglobin (g/dL)	14.6990 ± 1.55235	14.4015 ± 1.55235 (13.0–17.0)	0.196
Hematocrit (%)	43.9982 ± 4.75811	41.3615 ± 4.75811 (39–54)	<0.0001 **
Neutrophils (%)	34.5017 ± 11.55805	47.3740 ± 11.55805 (42.0–85.0)	<0.0001 **
Monocytes (%)	15.5553 ± 6.24152	4.9931 ± 6.24152 (0.0–10)	<0.0001 **
Lymphocytes (%)	50.0995 ± 10.21567	47.8129 ± 10.21567 (20–40)	0.183
Platelets (×10^9^ Cell/L)	234.8175 ± 39.54833	250.5846 ± 39.54833 (150–500)	0.130
**Oxidative stress biomarkers**			
Malondialdehyde (µmol/L × 10^−6^)	1.366 ± 0.11753	0.77 ± 0.08554 (0.36–1.24)	<0.0001 **
Superoxide dismutase [U/uL]	48.33 ± 1.0416	34.08 ± 1.0003 (13–23.7)	<0.0001 **

Data are presented as mean (±SD). MDs = motorbike drivers. Student’s *t*-test was used to compare means of blood parameters between categories of subjects. Significant correlation at a *p*-value less than 0.05. ** Significant correlation at a *p*-value less than 0.001.

**Table 6:** Changes in hematological parameters and variations in oxidative stress blood markers in motorbike drivers in Douala.

Oxidative Stress Markers		Red Blood Cell	Hemoglobin	Hematocrit	Neutrophils	Monocytes	Lymphocytes	Platelets	Number of Subjects
**SOD [U/uL].**	R Pearson	−0.086	−0.031	0.059	−0.298 **	0.378 **	0.101	−0.021	191
	*p* value	0.236	0.666	0.420	<0.0001	<0.0001	0.165	0.776	191
**MDA (µmol/L × 10^−6^)**	R Pearson	−0.061	0.019	−0.016	−0.161 *	0.161 *	0.098	−0.007	191
	*p* value	0.402	0.793	0.822	0.026	0.026	0.176	0.925	191

Bivariate correlation using Pearson’s test was used for assessing associations between oxidative stress markers and each category of hematological parameters. R = correlation coefficient. * Significant correlation at 0.05 level (bilateral). ** Significant correlation at 0.01 level (bilateral). SOD: superoxide dismutase, MDA: malondialdehyde.

**Table 7:** Correlation between exposure factors and changes in oxidative stress markers.

Oxidative Stress Markers		Place of Residence	Place of Work	Work Experience (Years)	Average Working Time (h/day)	Number of Subjects
**SOD [U/uL].**	t	−5.577 **	−0.812	1.596	−0.428	191
	*p* value	0.0001	0.418	0.112	0.669	191
**MDA (µmol/L × 10^−6^)**	t	−2135 *	−1077	0.687	0.931	191
	*p* value	0.034	0.283	0.493	0.353	191

Linear regression using ANOVA test was used for assessing associations between oxidative stress markers and each category of exposure factors. * Significant correlation at 0.05 level (bilateral). ** Significant correlation at 0.01 level (bilateral). SOD: superoxide dismutase, MDA: malondialdehyde.

**Table 8:** Influence of exposure factors on the changes in oxidative stress markers after adjustment for age, level of education, body mass index (BMI), smoking, and use of domestic gas, firewood, and coal.

Exposure Factors	MDA t (*p*-Value)	SOD t (*p*-Value)	Number of Subjects
Mean age	0.506 (0.613)	0.062 (0.951)	191
BMI	0.964 (0.336)	1.509 (0.133)	191
Categorized age	1.288 (0.199)	0.956 (0.340)	191
Level of education, n (%)	−0.606 (0.545)	−0.451 (0.653)	191
Place of residence	−2.195 (0.029)	−4.901 (<0.0001)	191
Place of work	−0.678 (0.499)	−0.633 (0.528)	191
Duration of work (per year)	−0.038 (0.970)	1.091 (0.277)	191
Daily duration of exposure (per hour)	1.057 (0.292)	0.158 (0.875)	191
Consumption of alcohol	1.183 (0.238)	−1.124 (0.263)	191
Domestic gas	0.029 (0.977)	−1.497 (0.136)	191
Firewood	−0.466 (0.641)	−0.384 (0.702)	191
Coal	−0.790 (0.431)	−0.746 (0.457)	191

Linear regression analysis was used for assessing associations between sites for the time of exposure (in years and hours), after adjustment for age, level of education, body mass index (BMI), consumption of alcohol, and use of domestic gas, firewood, and coal for each category of oxidative stress markers. Significant correlation at a *p*-value less than 0.05. Significant correlation at a *p*-value less than 0.001. t = regression coefficient.

**Table 9:** Correlation between respiratory disorders and changes in oxidative stress markers among motorbike drivers.

Oxidative Stress Markers		Sinusitis	Runny Nose	Cold	Current Fever	Sore Throat	Number of Subjects
**SOD [U/uL].**	t	−0.527	0.937	2.200 *	0.164	0.734	191
	*p* value	0.599	0.350	0.029	0.870	0.464	191
**MDA (µmol/L × 10^−6^)**	t	0.789	−0.596	2.588 *	1.341	1.651	191
	*p* value	0.431	0.552	0.010	0.181	0.100	191
**Oxidative stress markers**		Dry cough	Wheezing	Chest discomfort	Breathlessness		Number of Subjects
**SOD [U/uL].**	t	1.818	0.486	0.572	1.942		191
	*p* value	0.071	0.628	0.568	0.054		191
**MDA (µmol/L × 10^−6^)**	t	2.174 *	0.750	−1.198	−0.420		191
	*p* value	0.031	0.454	0.233	0.675		191

Linear regression using ANOVA test was used for assessing associations between symptoms and each category of oxidative stress markers. t = regression coefficient. * Significant correlation at 0.05 level (bilateral). SOD: superoxide dismutase, MDA: malondialdehyde.

**Table 10:** Correlation between general discomfort and changes in oxidative stress markers among motorbike drivers.

Oxidative Stress Markers		Headache	Dizziness	Eye Irritation	Conjunctivitis	Sweating	General Tiredness	Nausea	Number of Subjects
**SOD [U/uL].**	t	1.561	−0.722	1.248	0.579	1.040	1.658	−1.261	191
	*p* value	0.120	0.471	0.214	0.563	0.300	0.099	0.209	191
**MDA (µmol/L × 10^−6^)**	t	0.630	0.597	0.208	−0.089	0.997	1.185	−0.284	191
	*p* value	0.530	0.551	0.835	0.929	0.320	0.238	0.777	191

Linear regression using ANOVA test was used for assessing associations between symptoms and each category of oxidative stress markers. t = regression coefficient, SOD: superoxide dismutase, MDA: malondialdehyde.

## Data Availability

Data is unavailable due to privacy restrictions.
